# “A new era in Menkes disease management: the disease-modifying impact of Zycubo (copper histidinate)”

**DOI:** 10.1097/MS9.0000000000005090

**Published:** 2026-05-14

**Authors:** Fakiha Sultana Malik, Tayyaba Abdullah, Areej Imtiaz, Fatima Iftikhar, Mahnoor Fatima, Hafiz Naweed Ahmad

**Affiliations:** a Nishtar Medical College, Nishtar Medical University Multan, Pakistan; b Shaheed Ziaur Rahman Medical College, Rajshahi Medical University, Bogura, Bangladesh; c Department of General Surgery, Nishtar Hospital Multan, Pakistan

Menkes disease (MD) is a life-threatening X-linked multisystem disorder of copper metabolism, characterized by progressive neurodegeneration, connective tissue abnormalities, and the distinctive presentation of “kinky” hair. The disorder exhibits marked clinical heterogeneity in its severity, ranging from the classical form, which is the most severe, to occipital horn syndrome (OHS), regarded as the mildest variant. The majority of affected individuals are male, with rare female cases typically associated with X–autosome translocations that preferentially inactivate the normal X chromosome[1]. MD results from mutations in the ATP7A gene, located on chromosome Xq13.3, which impair the normal function of the ATP7A copper-transporting protein[2]. Current estimates suggest that the overall incidence of MD is approximately 1 in 100 000–250 000 live births, although regional differences are evident. Australia demonstrates a higher prevalence, estimated at 1 in 50 000–100 000 births. In contrast, a large observational study reported lower rates: 1 in 300 000 in Europe and 1 in 360 000 in Japan[3]. This article was written in accordance with the TITAN guideline checklist 2025[4].

The clinical manifestations of MD arise either directly from the dysfunction of copper-dependent enzymes or secondarily from the failure to incorporate copper into these enzymes adequately. For example, reduced cytochrome *c* oxidase activity impairs mitochondrial respiration, promoting central nervous system deterioration and ataxia. The clinical features include prolonged jaundice, hypothermia, hypoglycemia, feeding problems, kinky hair, dysmorphic facial features, and epileptic seizures[5].

Neonatal diagnosis of MD and early treatment may improve clinical outcomes[6]. Although rare, the rapid progression of neurological impairment and difficulties in prompt diagnosis underscore the meaningful healthcare implications of MD.

Zycubo (copper histidinate), approved by the FDA in January 2026, is the first disease-modifying therapy for MD, offering a targeted option to manage this rare condition. It is administered via intramuscular injection, with dosing adjusted according to patient weight and clinical needs. Its approval represents a significant advancement in therapy, providing a promising option for improving outcomes and quality of life for patients with MD[7].

In MD, mutations in the ATP7A transporter hinder intestinal copper absorption and its transport across the blood-brain barrier (BBB). Zycubo functions via a “Trojan horse” mechanism, using the LAT1 (SLC7A5) transporter to bypass impaired intestinal copper uptake and cross the blood–brain barrier, effectively functioning despite the failure of the ATP7A transporter. Zycubo (copper histidinate) overcomes this defect, ensuring effective copper delivery to both systemic and cerebral tissues[7]. Zycubo provides copper bound to histidine in a 1:2 molar ratio, forming a stable copper-histidine complex that bypasses ATP7A-dependent transport. Instead of the usual copper transporters CTR1 or ATP7A, this complex exploits the LAT1 (SLC7A5) amino acid transporter, allowing cellular entry and passage across the blood-brain barrier independently of the defective ATP7A. Mechanistic studies indicate that LAT1 mediates copper (histidinate) transport by switching from its normal antiport function to a uniport mechanism, allowing the copper payload to reach the brain and other copper-depleted tissues effectively[8]. Research on copper (II) histidinate complexes reveals that specific interactions with imidazole groups stabilize the copper and prevent oxidative damage from free copper, while also ensuring that copper remains available for essential enzyme activity, supporting normal cellular processes[9].

The approval of copper histidinate (Zycubo) by the FDA is based on two open-label, single-arm, single-center clinical trials. In the phase 1 and phase 2 clinical trials (NCT00001262), only newborn infants who had MD confirmed by biochemical or molecular grounds and did not show any neurological symptoms were included[10]. In the phase 3 clinical trial, subjects with classic MD, Occipital Horn Syndrome (OHS), or unexplained copper deficiency who had serum copper levels between 0 and 75 mg/dL were included[11]. In both studies, patients less than 12 months of age were administered 1450 mcg CuHis (250 mcg of elemental copper) in 0.5 mL of reconstituted product twice daily subcutaneously, and subjects who were older than 12 months of age were given the same dose once daily for 3 years[12]. For one of the single-arm studies, a comparison was made with historical control (HC) MD patients who did not receive copper treatment.

Primary and secondary efficacy analyses of patients born after 1999 with a severe ATP7A genotype mutation, who were divided into CuHis early treatment (CuHis-ET) versus HC early treatment (HC-ET) and CuHis late treatment (CuHis-LT) versus HC late treatment (HC-LT), showed statistically significant improvement in overall survival. A 75% reduction in the risk of death was observed in the primary efficacy analysis, with median overall survival of 177.1 and 16.1 months in ET subjects compared with HC subjects, respectively. Similarly, the secondary efficacy analysis showed a 75% reduction in mortality risk in the LT subjects compared with 17 HC subjects, with median overall survival of 62.4 and 17.6 months, respectively. Different sensitivity analyses were performed, which showed consistent results whether a severe ATP7A variant type or premature birth was present or not. Early treatment subjects who completed the treatment for a period of 3 years remained more active compared to subjects in the late treatment group, some of whom required feeding and respiratory support[13].

Although the single-arm design of the trials may be considered a limitation, it is scientifically reasonable here considering the rare nature of the disease[14]. The absence of randomization, the lack of a comparator arm, and reliance on historical control can introduce potential selection and confounding bias in the results. The single-center design limits the generalizability of the findings to other healthcare settings. These methodological limitations should be kept in mind when interpreting the observed survival benefits.

Treatment with Zycubo is associated with a spectrum of adverse reactions that reflect the inherent severity of MD. Clinical trials have shown that the most frequent adverse reactions, occurring in over 10% of patients, were respiratory infections, including pneumonia (30%) and viral infections (27%), as well as respiratory failure (23%), seizures (23%), and bacterial infections (20%)[15]. As these complications are characteristic of this disease, they may not result exclusively from Zycubo therapy. Similarly, complications such as bladder diverticula are recognized features of the disease itself rather than pharmacological effects of Zycubo.

Patients with MD often exhibit renal immaturity that predisposes them to an increased risk of copper accumulation and toxicity, which can manifest as renal tubular injury or hepatic dysfunction, particularly in ATP7A-related phenotypes, emphasizing the necessity for close surveillance and dose adjustments. Additionally, patients with MD inherently exhibit oxidative abnormalities due to disrupted copper-dependent enzymatic pathways[16]. Complications such as bladder diverticula are inherent to the natural progression of MD. These data highlight the need for close clinical supervision and early clinical action to mitigate potential complications[15]. Although Zycubo is the current standard of care, further research into other copper transporters, such as CTR1, is also expanding our understanding of copper metabolism disorders and may offer new therapeutic targets.

Prior to the approval of copper histidinate as a disease-modifying therapy, therapeutic options for MD were scarce and did not meaningfully modify its fatal natural history. Management was primarily supportive, including seizure control, nutritional support, and management of recurrent infections, while copper supplementation efforts were limited by defective ATP7A-mediated copper transport[6]. Early clinical studies tested parenteral copper histidine, which was adopted as the main therapy. These studies showed that starting therapy in the neonatal period, before neurological symptoms developed, can increase survival and partially preserve neurodevelopment. Treatment outcomes, however, vary and depend on the timing of therapy and the severity of the ATP7A mutation, with delayed treatment offering little benefit^[^1,17^]^. Despite these advances, inconsistent central nervous system copper delivery and the lack of standardized protocols kept copper therapy experimental for decades, emphasizing the unmet need that persisted until recent therapeutic developments.

The approval of Zycubo (copper histidinate) marks an important therapeutic development in the management of MD, providing the first therapy designed to alter its otherwise fatal progression. By bypassing defective copper transport mechanisms, Zycubo ensures effective delivery of copper to systemic and cerebral tissues, highlighting the potential importance of early diagnosis and intervention. This therapy has been associated with improvements in survival, while neurological outcomes and long-term safety remain insufficiently defined. In addition to its direct clinical benefits, the availability of Zycubo illustrates the evolving role of precision-based approaches in the diagnosis and treatment of rare genetic disorders. It highlights the potential of precision medicine, stresses the importance of early diagnosis, and emphasizes ongoing monitoring to optimize outcomes. Zycubo represents a meaningful therapeutic option, although its long-term impact remains to be fully defined.

## Ethical approval

Not applicable.

## Consent

Not applicable.

## Data Availability

Not applicable to this study.

## Guaranor

Mahnoor Fatima.
